# Transition to retirement impact on smoking habit: results from a longitudinal analysis within the Survey of Health, Ageing and Retirement in Europe (SHARE) project

**DOI:** 10.1007/s40520-023-02397-9

**Published:** 2023-04-17

**Authors:** Paola Bertuccio, Giacomo Pietro Vigezzi, Giansanto Mosconi, Silvano Gallus, Anna Odone

**Affiliations:** 1 Department of Public Health, Experimental and Forensic Medicine, Università degli Studi di Pavia, Via Forlanini 2, 27100 Pavia, Italy; 2 Ca’ della Paglia College, Fondazione Ghislieri, Piazza Collegio Ghislieri 5, 27100 Pavia, Italy; 3 Department of Environmental Health Sciences, Istituto di Ricerche Farmacologiche Mario Negri IRCCS, Via Mario Negri 2, 20156 Milan, Italy

**Keywords:** SHARE data, Retirement, Smoking, Life-course transitions, Longitudinal studies

## Abstract

**Background:**

In an ageing society, retirement impacts on behavioural risk factors and health outcomes should be carefully assessed. Scant evidence exists from longitudinal studies on the short- and long-term consequences of the transition to retirement on smoking habit.

**Methods:**

We conducted a longitudinal study based on the Survey of Health, Ageing and Retirement in Europe (SHARE) data from 27 European countries plus Israel collected in 2004–2020. To estimate relative risks (RR) and corresponding 95% confidence intervals (CI) for smoking status and intensity at seven time periods before and after retirement, we fitted adjusted generalised estimating equation (GEE) models for repeated measures.

**Results:**

We selected a cohort of 8998 individuals employed at baseline and retired at follow-up (median follow-up time: 9 years; maximum: 16 years). As compared to the year of retirement, the RR of smoking was 1.59 (95% CI 1.44–1.76) at 10 years or more before retirement, 1.35 (95% CI 1.25–1.46) from 5 to 9 years before retirement, and 1.18 (95% CI 1.10–1.27) from 1 to 4 years before retirement. Smoking steadily decreased after retirement, being 0.94 (95% CI 0.87–1.01) from 1 to 4 years after retirement, 0.76 (95% CI 0.69–0.84) from 5 to 9 years, and 0.58 (95% CI 0.46–0.74) 10 years or more after retirement. In smokers, the estimated number of cigarettes smoked/day decreased from about 27 cigarettes/day at 10 years or more before retirement to 9 cigarettes/day at 10 years or more after retirement (*p* trend < 0.001).

**Conclusion:**

Longitudinal data suggest that lifestyles might favourably change with retirement. Further studies are needed to direct healthy ageing promotion policies better.

**Supplementary Information:**

The online version contains supplementary material available at 10.1007/s40520-023-02397-9.

## Introduction

Increasing life expectancy and decreasing mortality are globally causing rapid ageing of the population. The share of older people is growing, and it is estimated that people aged 60 years and older will double between 2020 and 2050, from 1 to 2 billions, accounting for more than one-fourth of the whole population in high-income countries and two-thirds in low- and middle-income countries by 2050 [1].

Nowadays, people live longer after retirement than before [2], with several implications at different levels, especially for health and healthcare systems. Retirement represents a major life-course transition [3] that can have both negative and positive effects on physical and mental well-being. On the one hand, the end of the working life usually involves the loss of daily routines, as well as a reduction in social interactions and economic security. On the other hand, leaving demanding jobs might enable more time for leisure activities and exercise, making life less frantic [4, 5]. An increasing number of studies have been focusing on the association between retirement transition and behavioural risk factors patterns, including smoking, alcohol drinking, physical activity, and dietary habits, to explore the mechanisms that drive the retirement effect on health [4, 6].

Among lifestyles, smoking is a major cause of premature ageing [7]. It constitutes one of the most relevant modifiable risk factors for many chronic diseases [8], also placing a significant load on health systems [9]. Investigating the association between retirement and smoking can help better understand key aspects underpinning health outcomes in people transitioning from employment to retirement and thereafter. Data on retirement changes represent a unique opportunity to collect valuable evidence so as to target and time preventive interventions at an older age. However, data on this association are scanty, and conclusions are inconsistent: a large part of the available evidence on the topic is derived from cross-sectional studies [10, 11] or relies on longitudinal studies with short-term follow-ups [12, 13].

To provide much-needed evidence on the impact of retiring on smoking status over a more extended period, we used data from the Survey of Health, Ageing and Retirement in Europe (SHARE) project, the largest European multidisciplinary panel study, involving 27 European countries plus Israel with the scope to investigate the effects of health, social, economic and environmental policies over the life-course of adults aged 50 years or older [14]. In particular, taking advantage of the SHARE longitudinal panel data, our study aims to investigate and quantify the impact of the transition to retirement on smoking habit, focusing on pre- and post-retirement time windows, both from short- and long-term perspectives, among a large cohort of European retirees.

## Methods

### Study design and data source

We conducted a longitudinal cohort study using individual-level SHARE data. The protocol, study design and all study-related details are provided elsewhere [14, 15]. In brief, the SHARE project is based on a comprehensive set of databases, including cross-sectional and longitudinal individual-level data from 27 European countries plus Israel, collected in biannual waves since 2004, on current characteristics and behaviours, as well as retrospective life histories. SHARE data are collected during computer-assisted personal interviews (CAPI) through a questionnaire covering multidisciplinary fields, such as health, economic, social, and family dimensions, based on an ex-ante harmonised design to allow cross-country comparisons. All issues related to the data generation process are conducted following rigorous criteria [14, 16]. Moreover, ex-post harmonisation is adopted due to some internationally highly diverse country-specific variables and measurements [16, 17].

### Data linkage

Through a record-linkage procedure, we pooled individual-level SHARE data of waves 1 to 8 covering the period 2004–2020. In detail, for each wave, we merged three released databases, including information on sociodemographic characteristics (module DN), behavioural factors (module BR), and employment and pension variables (module EP). In addition, we integrated three other databases, including the so-called "generated variables" (i.e., gv_isced, gv_isco, and gv_health databases), to retrieve the harmonised data on education, occupation, and health indicators. In all the databases, the variable *mergeid* is the key, a unique and non-changing person identifier for all the waves. By merging longitudinal micro-data from all the waves 1 to 8, we constructed a cohort of European adults aged 50 years or older employed at baseline and transited to retirement at follow-up.

### Variables of interest

The exposure variable of our analysis was the time (in years) before and after retirement, computed as the difference between the year of retirement and the year of the interview. Time before and after retirement was split into seven periods: 10 years or more before retirement, from 5 to 9 years before retirement, from 1 to 4 years before retirement, the year of retirement (i.e., time 0), from 1 to 4 years after retirement, from 5 to 9 years after, and 10 years or more after retirement. The year of retirement was considered as the reference category.

The primary outcome of interest was smoking status at the time of the interview as a binary variable (1: current smoker; 0: non-smoker). The secondary outcome was the number of cigarettes smoked per day at the time of the interview, considering only the sub-group of individuals who declared to have smoked during the study period (i.e., ever-smokers).

A set of other variables was also considered as covariates: geographical area, sex, age group (50–54 years, 55–59 years, 60+ years), marital status (married/registered partnership, divorced/widowed, never married), educational level, occupational category, the presence of at least one chronic disease (yes, no). Educational level was classified according to the International Standard Classification of Education (ISCED) into three levels: low (ISCED levels: 0–1), intermediate (ISCED levels: 2–4) and high (ISCED levels: 5–6). For occupation, we considered the ten major groups of the International Standard Classification of Occupations (ISCO) [18]: 1) managers; 2) professionals; 3) technicians and associate professionals; 4) clerical support workers; 5) services and sales workers; 6) skilled agricultural, forestry and fishery workers; 7) craft and related trades workers; 8) plant and machine operators and assemblers; 9) elementary occupations; 10) armed forces. According to the World Bank classification [19], European countries were aggregated into the following geographical areas: North (including Denmark, Finland, and Sweden), West (Austria, Belgium, France, Germany, Luxembourg, the Netherlands, and Switzerland), South (Cyprus, Greece, Italy, Malta, Portugal, and Spain), East (Bulgaria, Croatia, Czechia, Estonia, Hungary, Latvia, Lithuania, Poland, Romania, Slovakia, and Slovenia), plus Israel.

### Statistical analysis

We fitted a generalised estimating equation (GEE) for binomial outcome with a log link function to estimate the probability of being smokers at seven different time periods (in years) before and after retirement. The model was used to estimate the relative risk (RR) of being a smoker and the corresponding 95% confidence intervals (CI) using the "year of retirement" as the reference category. A GEE model with negative binomial distribution and log link function was used to model the average number of cigarettes smoked per day at different time periods before and after retirement. GEE models allow the specification of a within-subject correlation structure, thus accounting for the repeated measures collected for each survey participant during the different waves. A set of covariates was included in the GEE models: geographical area, sex, age (continuous), marital status (married/registered partnership, divorced/widowed, and never married), educational level (low, intermediate, high), occupation (ISCO major categories) as baseline covariates, and the presence of at least one chronic disease (yes, no) as a time-varying covariate.

To verify the presence of potential effect modification or confounding on the associations, we conducted stratified analyses according to strata of geographical area, sex, baseline age group (50–54 years, 55–59 years, 60+ years), educational level (low, intermediate, high), occupational category (ISCO major groups 1 to 5 as non-manual workers, 6 to 9 as manual workers), and age at retirement (less than country-specific median, equal or greater than country-specific median).

Finally, we performed the following sensitivity analyses to estimate the associations for current smoking status: 1) we performed the overall analysis selecting only the 2606 retirees who already smoked at baseline; 2) we performed the overall analysis excluding the sub-group of 656 retirees who declared to receive a disability pension.

## Results

**Fig. 1:**
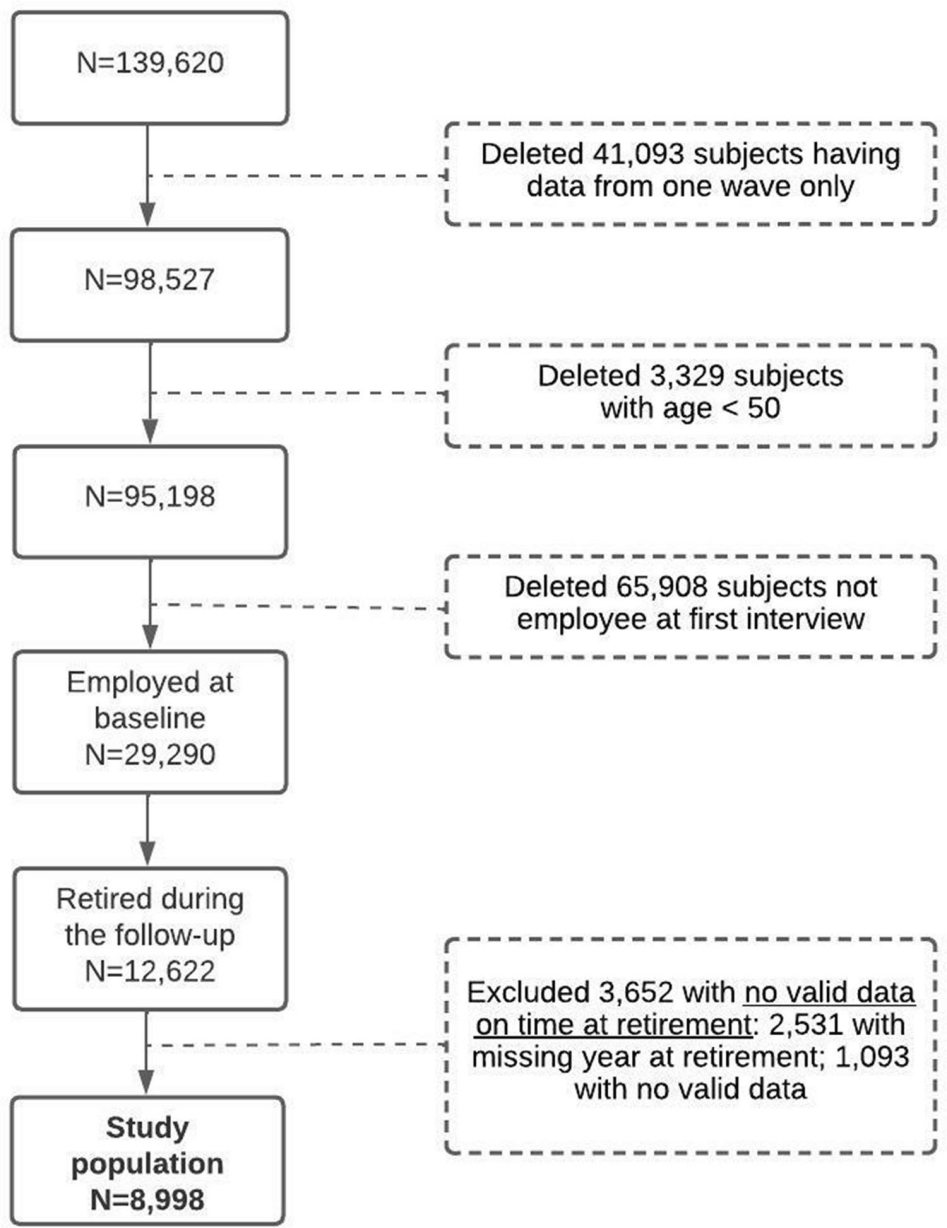
Flowchart of the study cohort selection

The inclusion and exclusion criteria of the study cohort are shown in Fig. 1. By merging data from SHARE waves 1 to 8, starting from a total of 139,620 individuals participating in at least one wave, we selected a cohort of 8998 individuals aged 50 or older who self-declared to be "employed" at baseline (i.e., their first interview) and retired at follow-up. The numbers of individuals from each of the 28 countries are detailed in Supplementary Figure S1. The age distribution at retirement for each country is given in Supplementary Table S1. The maximum follow-up time was 16 years, and the median was 9 years.

Table 1 reports the distribution of the overall cohort according to the geographical area and selected baseline characteristics. Fifty-three percent of the overall study cohort were males; 42.6% were between 55 and 59 years old (mean age: 57 years; standard deviation: 4.2); 56.7% and 31% had intermediate and high education levels, respectively; and 83.9% were married. Professionals were the most frequent occupation group (15.5%), followed by technicians and associate professionals, clerical support workers, services and sales workers (about 13.6%), and managers (10.5%).

**Table 1:** Distribution of the overall study population aged 50 or more (*n* = 8998) according to European geographical area and selected baseline characteristics, 2004–2017

	N	%
European area		
North	1626	18.07
West	3738	41.54
South	1480	16.45
East	1880	20.89
Israel	274	3.05
Sex		
Male	4797	53.31
Female	4201	46.69
Age group (years)		
50–54	2504	27.83
55–59	3831	42.58
≥ 60 (max: 83)	2663	29.60
Education level (ISCED)		
Low (0–1)	1044	11.60
Intermediate (2–4)	5102	56.70
High (5–6)	2787	30.97
Missing	*65*	*0.72*
Marital status		
Married/Registered partnership	7552	83.93
Divorced/Widowed	1229	13.66
Never married	107	1.19
Missing	*110*	*1.22*
Occupation (ISCO categories)		
Managers	944	10.49
Professionals	1397	15.53
Technicians and associate professionals	1230	13.67
Clerical support workers	1209	13.44
Services and sales workers	1223	13.59
Skilled agricultural, forestry and fishery workers	341	3.79
Craft and related trades workers	872	9.69
Plant and machine operators and assemblers	483	5.37
Elementary occupations	715	7.95
Armed forces	88	0.98
Missing	*496*	*5.51*

**Fig. 2:**
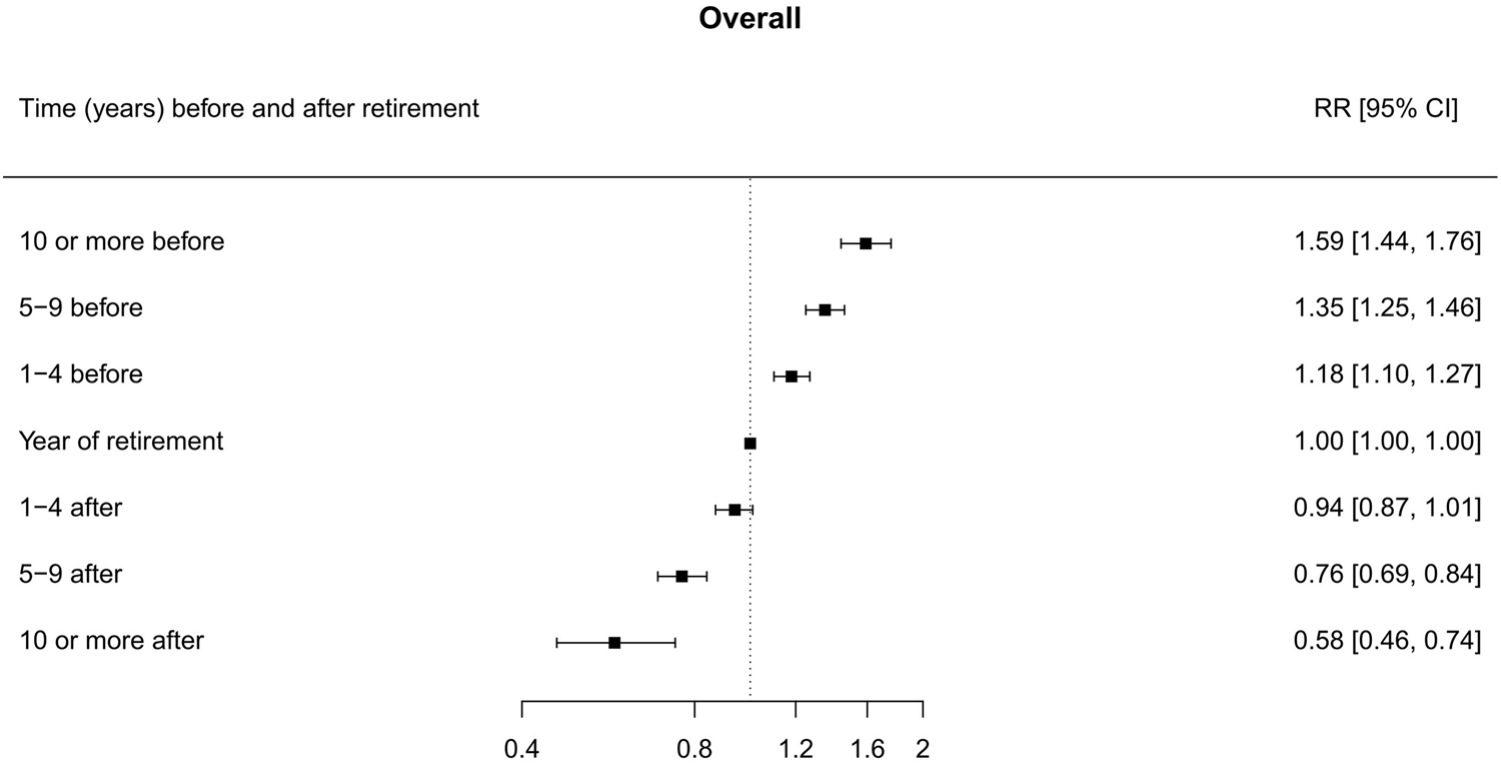
Forest plot of the relative risk (RR)* and corresponding 95% confidence intervals (CI) for the current smoking status (yes *vs* no) at seven different periods before and after retirement (reference category: the year of retirement). *Estimates were obtained from a GEE model for repeated measures, adjusted by geographical area, sex, age (continuous), marital status (married/registered partnership, divorced/widowed, and never married), educational level (low, intermediate, high), occupation (ISCO major categories) as baseline covariates, and the presence of at least one chronic disease (yes, no) as time-varying covariate

Figure 2 reports the RR of being a smoker in relation to the studied time periods before and after retirement. As compared to the year of retirement, the prevalence of smoking was 59% higher in the period 10 or more years before retirement (RR 1.59; 95% CI 1.44–1.76), 35% higher in the period from 5 to 9 years before retirement (RR 1.35; 95% CI 1.25–1.46) and 18% higher from 1 to 4 years before retirement (RR 1.18; 95% CI 1.10–1.27). The prevalence of smoking decreased by 6% from 1 to 4 years after retirement (RR 0.94; 95% CI 0.87–1.01), 24% from 5 to 9 years after retirement (RR 0.76; 95% CI 0.69–0.84), and 42% from 10 or more years after retirement (RR 0.58; 95% CI 0.46–0.74). Such a trend remained consistent in stratified analyses by geographical area, sex, baseline age group, educational level, occupational category, and age at retirement (Supplementary Figure S2). Similarly, estimates did not significantly change when we performed sensitivity analyses (data not shown).

**Fig. 3:**
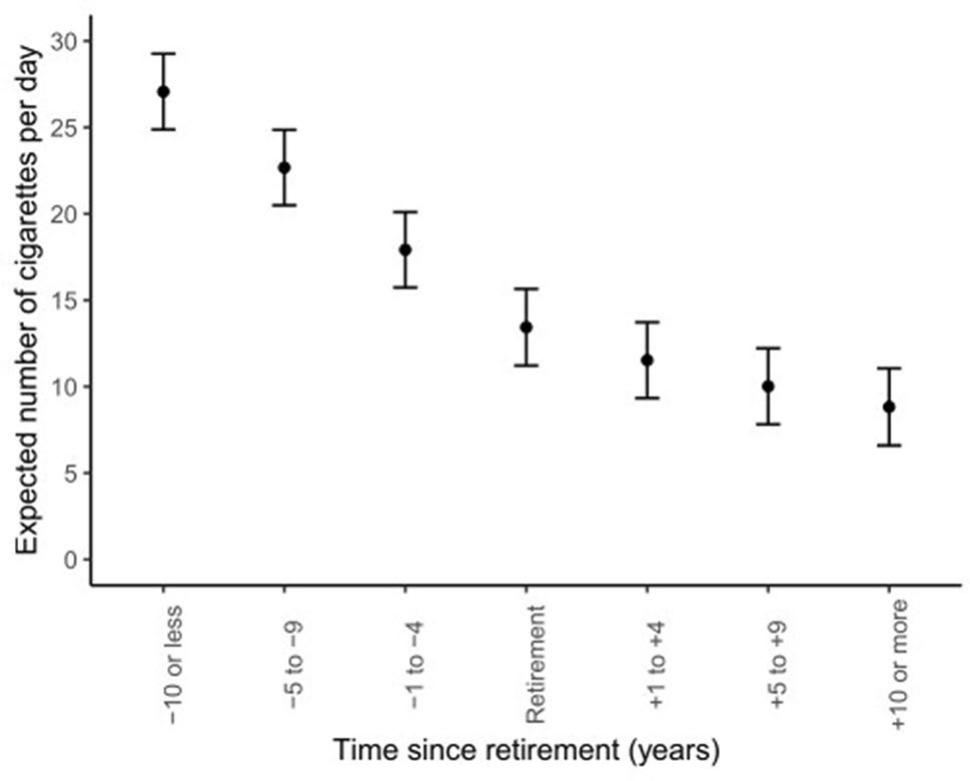
Predicted values* and 95% confidence intervals (error bars) of the number of cigarettes smoked per day at different time periods before and after retirement, among the ever smokers over the study period. *Estimated through a GEE model for repeated measures, adjusted by the following baseline covariates: geographical area, sex, age (continuous), marital status (married/registered partnership, divorced/widowed, and never married), educational level (low, intermediate, high), occupation (ISCO major categories), and the presence of at least one chronic disease (yes, no) as a time-varying covariate

In ever smokers, the average number of cigarettes smoked per day, as shown in Fig. 3, decreased when the time periods approached the year of retirement, from 27.1 (95% CI 24.9–29.3) cigarettes per day 10 years or more before retirement to about 13.4 (95% CI 11.2–15.7) cigarettes per day at the year of retirement. Predicted values continued to decrease after retirement with 11.5 (95% CI 9.3–13.7), 10.0 (95% CI 7.8–12.2), and 8.8 (95% CI 6.6–11.1) cigarettes smoked per day, from 1 to 4, 5 to 9, and 10 years or more after retirement, respectively, (*p* for trend < 0.001).

## Discussion

We investigated short- and long-term effects of the transition to retirement on smoking habit—one of the major behavioural risk factors—in a large longitudinal European cohort of nearly 9000 individuals who transited from employment to retirement during the study period. We report a significant linear trend in reducing smoking habits progressively over a long life-course period before and after retirement. Considering the period of 10 years or more before retirement, the prevalence of smoking was about 60% higher compared to the year of retirement. It decreased progressively, and the trend remained consistent after retirement, up to over 40% reduced prevalence 10 years or more after retirement. Along the same lines, among the ever smokers, the number of cigarettes smoked per day decreased from around 27 cigarettes/day 10 years or more before retirement until about 9 cigarettes/day 10 years or more after retirement. Our estimates remained consistent when considering strata of geographical area, sex, baseline age group, education, occupation, and age at retirement. Besides an intrinsic age effect, other mechanisms might be involved in the causal relationship between retirement and smoking prevalence.

The reduction in smoking we observed after retirement might be explained by several factors acting on the association. First, stress factors and peer pressure, among the most important reasons for starting and keeping tobacco consumption [20, 21], substantially decrease along with exiting employment. As stated by a recent systematic review, job-related stress may lead to intensifying smoking [22]. It is possible that leaving demanding and stressful work aids many people in cutting down or quitting smoking [20].

Second, age and associated diseases might play a role, which is why we adjusted our model. As reported in previous surveys, age and incoming chronic diseases might act as relevant drivers of smoking cessation, suggesting that smokers generally quit tobacco to avoid future health problems or due to current health conditions [23]. Yet, in support of the independent effect of retirement on smoking status, our sensitivity analysis, excluding disability pensions as a proxy of bad health, confirmed the reduction of smoking habits after the transition. Indeed, our findings are coherent with the prevalence trends of tobacco smoking across Europe according to age groups. A large cross-sectional study conducted in 12 European countries [24] showed a decreasing trend in smoking habits with increasing age. However, the same study found higher smoking prevalence among men, less educated individuals, and those with a low socioeconomic status (SES). In agreement with other studies [25, 26], geographical differences were shown in smoking prevalence, with favourable patterns and trends in Northern Europe while unfavourable in Eastern countries. In contrast, we did not find any significant heterogeneity in smoking across geographical areas or SES levels, maybe because of the relative homogeneity of our selected sample.

Extensive evidence exists on the impact of retirement on behavioural determinants of health and health status, mainly in the social and economic fields [27, 28]. Mounting literature focuses on the mechanisms driving the health effects of retirement, such as changes in lifestyles, including smoking, alcohol drinking, dietary habits, and physical activity after retirement [4, 13]. Changes in lifestyles when transitioning to retirement are related to both individual and environmental factors, consisting, on the one hand, of demographic aspects, characteristics of retirement (e.g., type and intention), those of employment (e.g., time pressure, workload and physical demand), access to socioeconomic resources, along with personality characteristics, such as expectations and fears about the consequences of this crucial turning life point [29], and, on the other hand, of contextual factors, such as the retirement policies.

Given the complex conceptual framework behind retirement transition, evidence is based on different methodological approaches, definitions, populations and outcomes, thus leading to inconsistent conclusions [12, 30]. Looking at the literature on the relationship between retirement and smoking, a recent systematic review summarised the currently available evidence [12]. In agreement with our results, some studies showed that retirement was associated with a lower risk of smoking [31–37], whereas other studies did not report any significant association [38–43], and one found retirement to be associated with an increased risk [10]. In particular, three studies analysed SHARE data from a longitudinal perspective, even though they used approaches other than ours.

Considering the individual characteristics, one of the included studies explored health behaviour changes among older people during their transition from employment to retirement addressing the differences according to the baseline health behaviours, beyond other sources of individual heterogeneity such as gender, European geographic area, and job type [36]. This study suggested that older adults who did not smoke at baseline tended to avoid smoking after retirement, and those who smoked tended to smoke less, as confirmed by our sensitivity analysis.

Moreover, individual behaviours may directly affect the health and behaviours of other household components, positively or negatively [44, 45]. Based on this assumption, another SHARE study, including data from 19 European countries, focused on household context by assessing the partner's retirement impact on the health behaviours and the health status of the other partner [43]. Retirement of a partner increased the number of cigarettes smoked only among the already smokers, partially explaining the additional finding of a negative impact of spousal retirement on individual health status.

Regarding sex, men and women may differentially cope with retirement transition consequences [46, 47], likely due to the different roles they may play at work and home [28]. However, our results did not suggest differences in the estimates according to sex.

About employment, an earlier study based on the SHARE data from 10 European countries investigated the retirement effect on behaviours, including smoking, linked to several individuals' job characteristics. They found a heterogeneous retirement effect on smoking according to occupation type, showing that individuals classified as blue collars but not white collars were less likely to smoke after retirement [35]. Conversely, one study from the USA, based on the longitudinal panel Health and Retirement Study (HRS), found an increase in the probability of smoking after retirement across different occupational categories. However, the study did not assess the long-term effects of retirement as in our analysis, which showed a significant and more relevant reduced smoking prevalence since 5 years after retirement. Smokers may continue to smoke immediately after retirement but could quit for a longer period after they set new daily routines [37].

Coming to the role of the retirement process, its effect on health behaviours can also be partially influenced by retirement intentions [48]. A panel study from the Netherlands investigated the retirement effect on smoking habits among voluntary and involuntary retirees [40]. Workers who retired involuntarily reported a lower probability of reduced smoking than workers who resigned voluntarily. These findings support the conclusion that people might use tobacco and alcohol as a coping strategy for the emotional stress brought on by an unanticipated leave from paid employment [40].

Taking into consideration the contextual environment, individual expectations, actions and decisions regarding retirement may vary according to the pension policies and socioeconomic context of the country of origin and, therefore, could differentially impact health behaviours, particularly smoking status. However, in contrast with our findings, studies conducted in similar geographical areas showed substantial heterogeneity [12], indicating that other factors may play a role. The mixed results of retirement impact on smoking available in the literature can be likely due to the different contexts, data and methodological approaches used.

Findings from this study should be interpreted in light of both strengths and limitations. One limitation is that retirement status and year of retirement, as well as the study outcomes and covariates, are based on self-reported information and are consequently subject to recall bias and miss-reporting. Nevertheless, evidence from our study is supported by data on smoking prevalence trends by age in Europe [24]. We did not consider differences in retirement policies and smoking bans across countries that may have affected both exits from employment and smoking habits during the life course of the study participants. Since the study cohort was selected following subjective criteria, its representativeness and the generalisability of our results can be limited.

Despite these limitations, our findings are based on a high-quality representative data source for studying multiple factors linked to the health and environmental conditions of individuals aged 50 or more in Europe. To our knowledge, this is the first work that used an epidemiological approach to carry out a longitudinal cohort analysis. This allowed us to investigate the most extended possible follow-up period within the SHARE project, by pooling data from all the cross-sectional waves. Therefore, we could derive estimates at different time periods before and after retirement (i.e., pre and post) as short- and long-term effects, providing unique findings that contribute to exploring the "honeymoon" retirement effect from a long-term perspective and monitoring its temporal evolutions. The cohort study design, used for investigating the retirement transition impact on smoking habit, did not require a control group which otherwise implies several issues in terms of comparisons and matching. Moreover, by performing a repeated measures analysis for longitudinal individual data using GEE models, we captured all the available information collected across the waves, with several advantages in terms of efficiency and uncertainty of the estimates. Finally, to reduce potential confounding, we adjusted our estimates for the main confounding factors, including covariates that change over time, such as the presence of chronic diseases.

The transition to retirement represents a crucial stage in life, followed by social, psychological, and physical changes that significantly impact health. Depending on whether retirement is perceived as a loss or a relief, it may have a dual impact on retirees' lifestyles [49, 50]. While retirement could positively act on behavioural patterns thanks to fewer work-related stressful factors and increased leisure time, retired individuals can also experience negative feelings due to loss of social and intellectual interactions, loss of daily routines, possible loss of income as well as a decrease in work-related physical activity, thus accelerating a worsening in health during ageing.

Regarding public health policies, welfare and health interventions might substantially contribute to shifting the retirement transition impacts towards a positive new balance for the individual. Changes in established habits and behaviours may be more challenging in older age, and not all authors agree that health promotion and prevention initiatives can still find fertile ground at this stage of life [51]. Although evidence on the topic is still conflicting, our findings suggested that large-scale behavioural changes could occur after retirement, specifically in reducing smoking habits. Therefore, this study supports the hypothesis that short and long times around retirement might offer an ideal opportunity to make relevant lifestyle adjustments and that people going through this phase may still be addressed by targeted, effective health promotion and primary prevention interventions.

About public policies, since the transition to retirement is likely to have a favourable effect on smoking habits independently from geographical and sociodemographic patterns, pension policies might broadly influence the health of various types of workers simultaneously, beyond differences in personal characteristics and socioeconomic context. Therefore, coordinating public health strategies with life course transitions, which frequently trigger lifestyle changes, might be particularly beneficial [52], for instance, introducing more flexibility in exiting from the labour force according to an individual's health status.

Concerning research, shared methodological standards and definitions, such as those used by SHARE, should be extensively adopted in the future to reduce heterogeneity and accumulate solid evidence. At the same time, special attention should be paid to further differentiating contextual and individual characteristics to disentangle and quantify the role of the mediators acting on the relationship between retirement and behavioural factors and, thus, identify specific disadvantaged groups.

In conclusion, based on an epidemiological approach, this is the first attempt to investigate the short- and long-term impact of the transition to retirement on smoking habit among a large European cohort of retirees, using the SHARE longitudinal panel individual data, suggesting a favourable effect, independently from geographical and individual sociodemographic characteristics. This encompasses an individual age effect but also indicates that other mediators and mechanisms can likely explain the causal relationship between retirement and smoking. Moreover, this effort will allow us to investigate the retirement transition impact on other lifestyles and potential health drivers [4, 13, 28], including alcohol drinking, dietary habits [53] and physical activity, as well as mental health indicators [54, 55], with the scope to provide more evidence on this topic and highlight the importance to target preventive actions to promote healthy ageing.

## Supplementary Information

Below is the link to the electronic supplementary material.

Supplementary file1 (PDF 781 KB)

## Data Availability

The datasets supporting the conclusions of this study are publicly available from SHARE Research Data Center (https://releases.sharedataportal.eu/) upon request.
