# Supplementary material for: Transition to retirement impact on smoking habit: results from a longitudinal analysis within the Survey of Health, Ageing and Retirement in Europe (SHARE) project
Source: Aging Clin Exp Res. 2023 Apr 17;35(5):1117–26. doi: 10.1007/s40520-023-02397-9 (PMC10149464; doi:10.1007/s40520-023-02397-9)

SUPPORTING MATERIALS

Manuscript's title: "Transition to retirement impact on smoking habit: results from a longitudinal analysis within the Survey of Health, Ageing and Retirement in Europe (SHARE) project".

Index

Supplementary Table S1. Distribution of age at retirement by country. 2

Supplementary Figure S1. Country and number of individuals (n) according to the calendar years in the study setting (2004-2020). 3

Supplementary Figure S2. Forest plot of the relative risk (RR)* and corresponding 95% confidence intervals (CI) for the current smoking status (yes *vs* no) at different time periods before and after retirement (reference category: the year of retirement) across strata of: geographical area (panel A), sex (panel B), age group (panel C), educational level (panel D), occupational category (panel E), and age at retirement (panel F)................. 4

Supplementary Table S1. Distribution of age at retirement by country.

Country	N	Minimum	25° percentile	50° percentile (median)	75° percentile	Maximum
Austria	431	52	59	60	62	72
Belgium	765	52	60	61	64	78
Bulgaria	31	55	61	62	63	64
Croatia	46	54	60	62	65	69
Cyprus	12	63	63	64	65	65
Czech Republic	624	52	59	61	62	72
Denmark	685	51	61	63	65	80
Estonia	504	51	62	63	67	85
Finland	54	55	63	63.5	65	69
France	837	53	60	61	62	76
Germany	609	53	62	64	65	75
Greece	501	52	61	65	67	78
Hungary	47	55	58	61	64	65
Israel	274	54	62	65	67	85
Italy	395	51	59	61	65	79
Latvia	20	61	63	63	64	80
Lithuania	28	61	62	63	64	64
Luxembourg	108	55	58	60	62	71
Malta	23	57	61	61	63	65
Netherlands	436	55	61	64	66	79
Poland	271	51	60	60	63	70
Portugal	54	53	60	62	65	66
Romania	29	53	59	61	62	68
Slovakia	53	50	60	62	62	66
Slovenia	227	51	58	60	61	71
Spain	495	52	61	64	65	80
Sweden	887	52	64	65	65	84
Switzerland	552	55	63	64	65	83

Supplementary Figure S1. Country and number of individuals (n) according to the calendar years in the study setting (2004-2020).

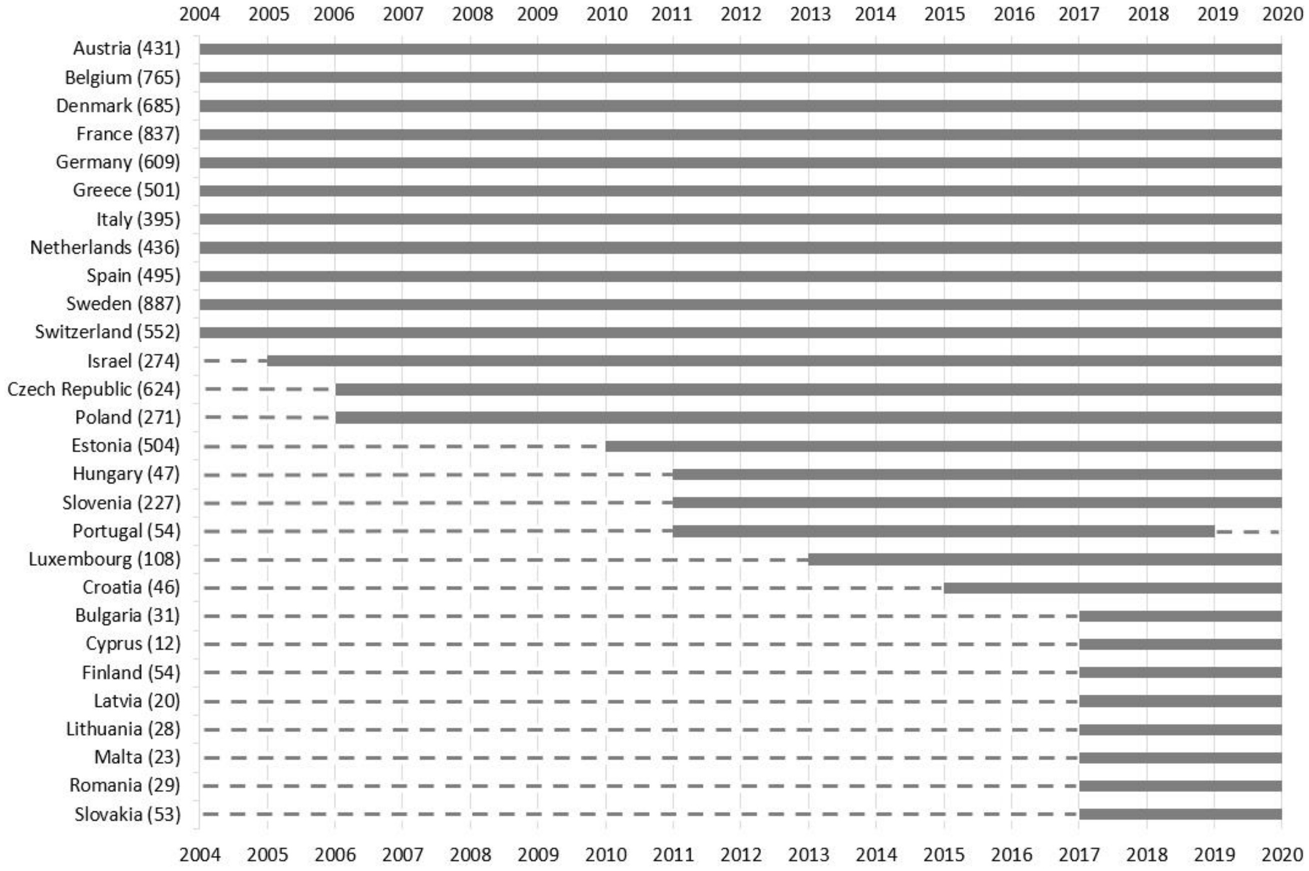

Supplementary Figure S2. Forest plot of the relative risk (RR)* and corresponding 95% confidence intervals (CI) for the current smoking status (yes *vs* no) at different time periods before and after retirement (reference category: the year of retirement) across strata of: geographical area (panel A), sex (panel B), age group (panel C), educational level (panel D), occupational category (panel E), and age at retirement (panel F).

Panel A

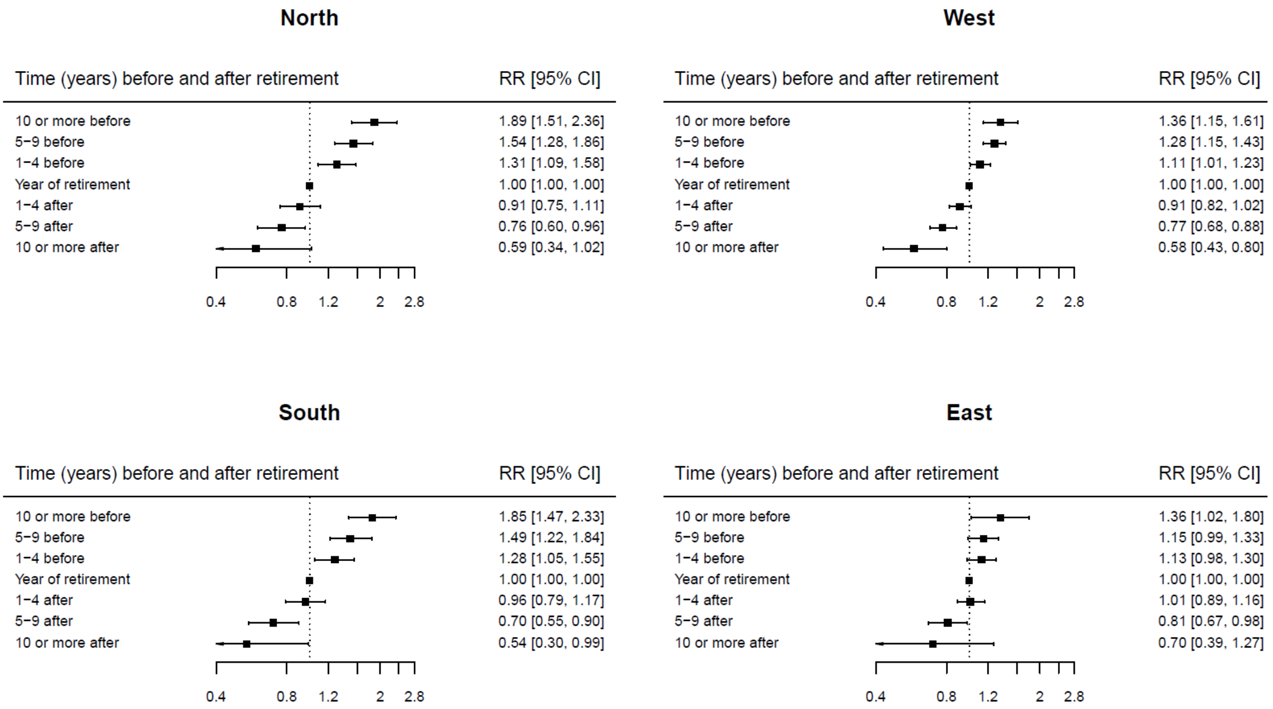

Panel B

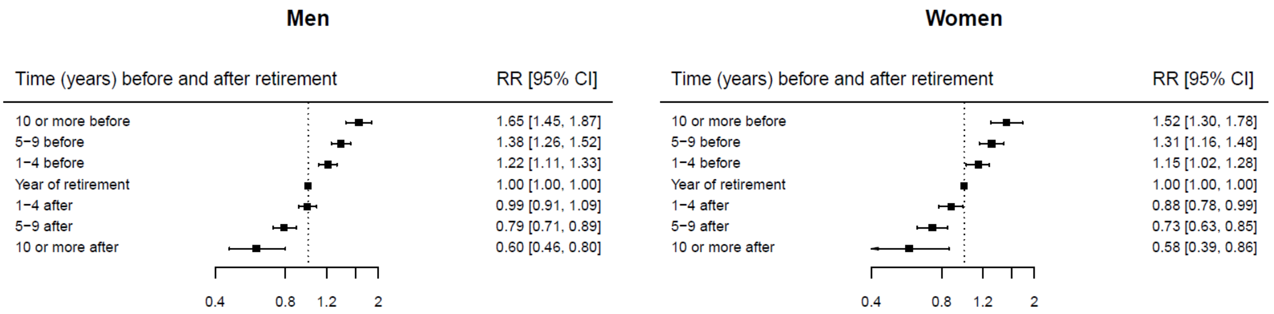

Panel C

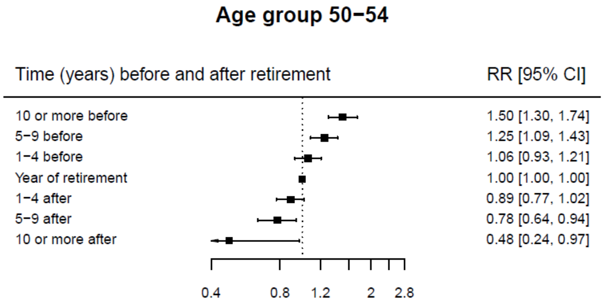

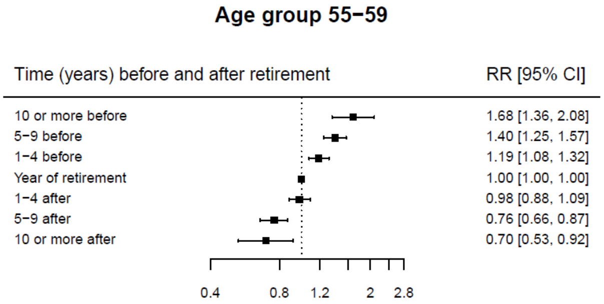

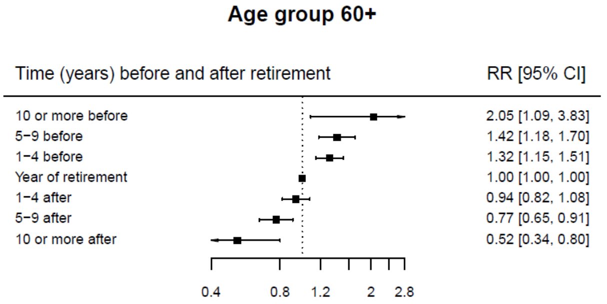

Panel D

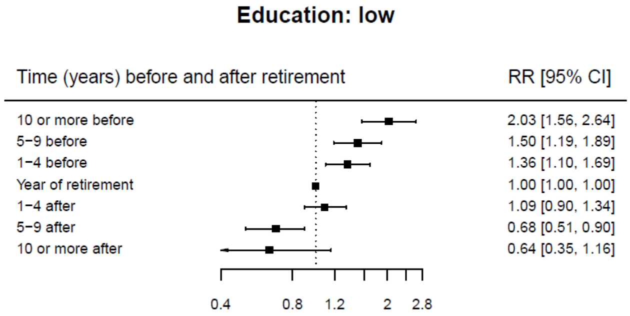

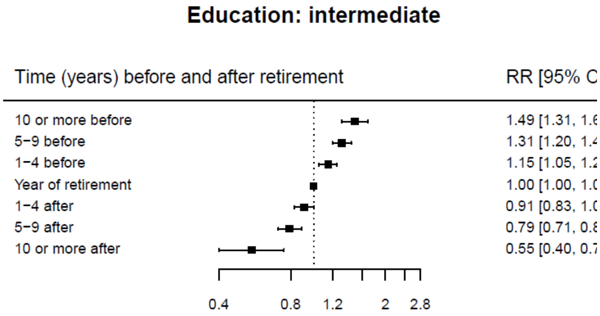

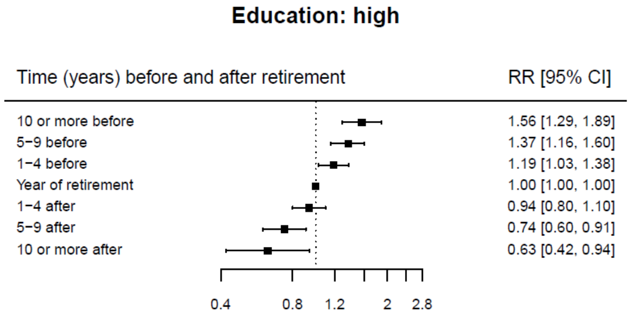

Panel E

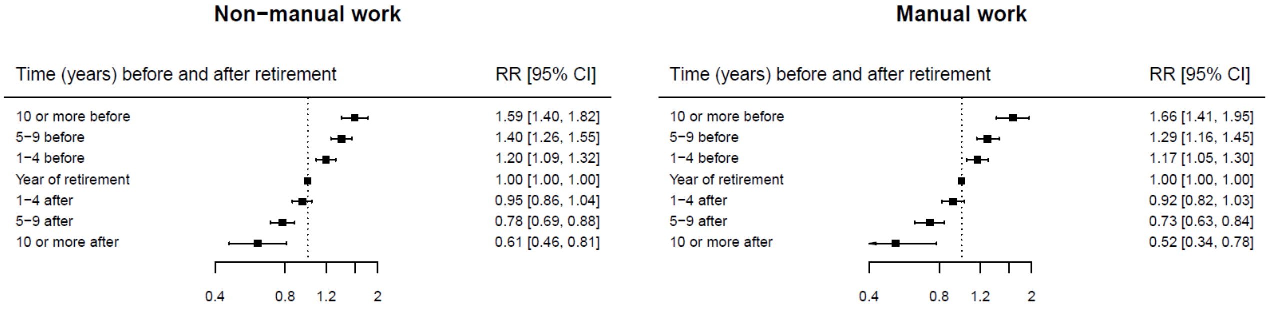

Panel F

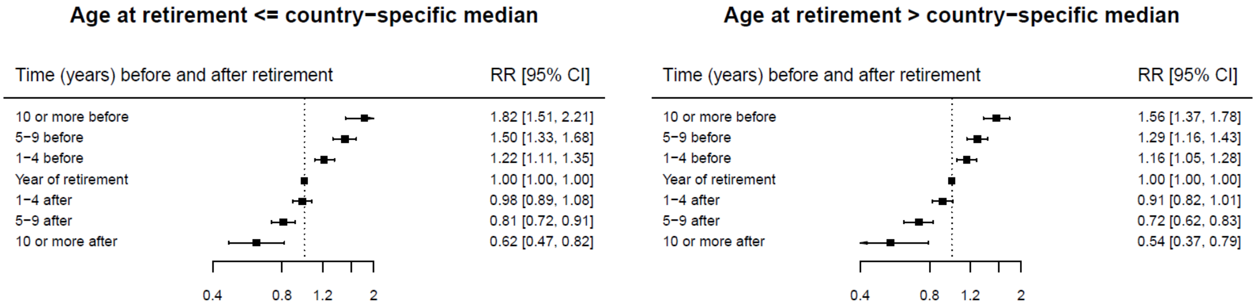

Supplement: Supplementary file 1 — Supplementary file1 (PDF 781 KB) [file 40520_2023_2397_MOESM1_ESM.pdf]
